# Argon laser peripheral iridoplasty for plateau iris associated with iridociliary cysts: a case report

**DOI:** 10.1186/1757-1626-1-368

**Published:** 2008-12-02

**Authors:** Ghee Soon Ang, Frank Bochmann, Augusto Azuara-Blanco

**Affiliations:** 1 Eye Clinic, Aberdeen Royal Infirmary, Foresterhill, Aberdeen, AB25 2ZN, Scotland, UK; 2 Department of Ophthalmology, Cantonal Hospital of Lucerne, 6000 Lucerne 16, Switzerland

## Abstract

**Introduction:**

Plateau iris is recognised as an important cause of primary angle closure glaucoma. The management of this condition generally comprises laser peripheral iridotomy and iridoplasty, to remove any component of relative pupillary block and to widen the iridotrabecular drainage angle respectively. However, plateau iris may be associated with multiple iris cysts at the iridociliary junction, which then presents diagnostic and management problems.

**Case presentation:**

We present a fifty-three year old Caucasian gentleman with plateau iris associated with peripheral iris cysts, in which the iridotrabecular angle did not widen despite having had both laser peripheral iridotomy and iridoplasty. The patient has remained asymptomatic over 12 months, and is under close follow-up to monitor for signs of glaucoma.

**Conclusion:**

Plateau iris with iridociliary cysts can be difficult to diagnose and manage. Ultrasound biomicroscopy should be performed on patients with appositional iridotrabecular angle closure on gonioscopy, especially if the angle closure is not relieved with either laser peripheral iridotomy or iridoplasty. Question marks can be raised as to the benefit of laser iridotomy when plateau iris without pupillary block has already been conclusively diagnosed on ultrasound biomicroscopy.

## Introduction

Plateau iris is now recognised as an important cause of primary angle closure glaucoma. Plateau iris may be associated with multiple iris cysts at the iridociliary junction. This case demonstrates the difficulties that can arise in the diagnosis and management of plateau iris in conjunction with peripheral iris cysts.

## Case presentation

A 53-year old Caucasian gentleman was referred to the Glaucoma Clinic by the community optometrist due to raised intraocular pressure (IOP). He was asymptomatic, and did not have any past medical history or family history of note. His distance vision was 6/6 unaided in both eyes, and his near vision was N5 bilaterally. 24-2 Humphrey visual fields were full. His Goldmann applanation IOPs were 18 mmHg in both eyes. Central corneal thickness measurements were normal at 544 microns in the right and 540 microns in the left. Anterior segment examination was unremarkable, with deep-looking anterior chambers and mild lens opacities. Both optic discs had healthy neuroretinal rims with a central cup-to-disc ratio of 0.3. The iris profile was smooth, with no atrophy, thinning, elevations or bulges. However, gonioscopy revealed bilateral narrow iridotrabecular angles with plateau iris configuration throughout the entire circumference, with the characteristic 'double-hump' of the iris seen on indentation. There were no areas of peripheral anterior synaechiae. He was therefore given the provisional diagnosis of narrow iridotrabecular angles with plateau iris configuration.

In order to confirm the diagnosis, neodymium:yttrium-aluminium-garnet (Nd:YAG) laser peripheral iridotomy was performed. The 'gush' of fluid and pigment from the iridotomy sites at the time of laser confirmed successful full-thickness iris penetration. At the next follow-up visit, repeat gonioscopy did not reveal any change in the iridotrabecular angle configuration despite the presence of a patent iridotomy in both eyes. Ultrasound biomicroscopy (UBM) of the angles was then performed, and confirmed the typical features of plateau iris – very narrow or apposed iridotrabecular angle, a flat or slightly convex contour of the iris, and absence of the iridociliary sulcus. In addition, multiple cysts were found at the iris root and iridociliary junction in many, but not all, meridians. The laser iridotomies were also confirmed to be patent in both eyes.

**Figure 1:**
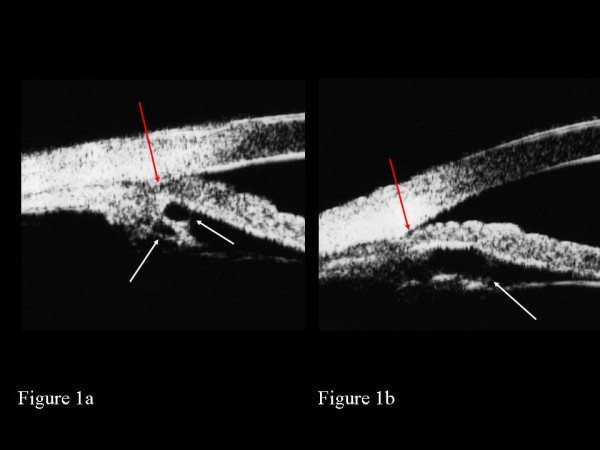
**Ultrasound biomicroscopy of plateau iris with iridociliary cysts**. Ultrasound biomicroscopy of the left eye showing iridociliary cysts (white arrows) with closed iridotrabecular angle (red arrow) at 9 o' clock (Figure 1a) and narrow iridotrabecular angle (red arrow) at 3 o' clock (Figure 1b) after both laser peripheral iridotomy and iridoplasty.

The decision was then made to perform argon laser peripheral iridoplasty, initially in the left eye. Using an Abraham iridectomy contact lens, 24 argon laser burns (500 micron spot size, 500 ms duration, 350 mW power) were placed circumferentially at the periphery of the iris as close to the limbus as possible. He was given a 1-week course of topical dexamethasone 0.1% qid to the left eye post-laser. On review 1 month later, gonioscopy still showed no change in the drainage angle configuration, with apposed or very narrow iridotrabecular angles. This was confirmed on UBM (Figures 1a and 1b). During that visit, his IOPs were measured at 26 mmHg in both eyes, without any topical medication. Since he was asymptomatic and had no signs of glaucoma damage, we elected to monitor him closely with a view to commencing treatment if required. As there had been no effect from laser iridoplasty in the left eye, this was not performed in the right eye. At the last clinic visit, his IOPs were 22 mmHg with apposed or very narrow iridotrabecular angles and healthy optic discs in both eyes. So far, he has not developed any symptoms of acute angle closure glaucoma over the past 12 months since diagnosis. He remains under regular review at the glaucoma clinic.

## Discussion

Plateau iris was first described in 1958. [1] However, it has only been relatively recently recognised as an important cause of primary angle closure glaucoma. Plateau iris configuration is defined as a narrow or closed iridotrabecular drainage angle, associated with a flat iris plane and a relatively normal anterior chamber depth. It is the anatomic appearance whereby the iris root, from its insertion point at the iridocorneal angle, initially angulates sharply forward and then curves centrally. Indentation gonioscopy reveals the characteristic 'double hump' appearance. Plateau iris syndrome occurs when the iridotrabecular angle remains closable despite the presence of a patent laser iridotomy or iridectomy, which would have removed any component of pupillary block. The mechanism for this is thought to be due to a large or anteriorly-positioned ciliary body pushing the peripheral iris against the trabecular meshwork. [2] Plateau iris tends to occur in females, aged from 30 to 50, and with a family history of angle closure glaucoma. The prevalence of plateau iris is unknown.

High-frequency ultrasound biomicroscopy (UBM) is a useful tool that allows assessment of the iris, iridotrabecular angle structures, ciliary body, and posterior chamber. In plateau iris, UBM typically shows iridotrabecular apposition, a flat or slightly convex contour of the iris, and absence of the iridociliary junction. [3] The anterior chamber depth is shallower than normal, although clinically it may appear relatively deep. [4] Current anterior chamber optical coherence tomography (OCT) technologies are not useful in these cases because they are unable to provide adequate images of the ciliary sulcus and processes.

Plateau iris may be associated with cysts in the peripheral iris root or ciliary body. [5] These cysts displace the peripheral iris anteriorly, thus giving rise to 'pseudoplateau iris'. These idiopathic peripheral iris root cysts usually arise from the iris pigment epithelial layer at the iridociliary junction. Iridociliary cysts occur more frequently in females, with a mean age of 33 years at diagnosis. [6] They are usually round or ovoid, and are centrally echolucent with thin hyperreflectile walls on UBM. [7] The prevalence of peripheral iris cysts causing pseudoplateau iris is unknown.

So what are the treatment options? Topical pilocarpine reduces IOP, and is effective for widening the iridotrabecular drainage angle in plateau iris by stretching and thinning the peripheral iris. [8] However, it can be poorly tolerated. It is also interesting to note that prolonged usage of topical miotics can result in secondary iris cysts. [9]

Unlike in pupil block, Nd:YAG laser peripheral iridotomy in plateau iris does not alter the width of the anterior chamber or the configuration of the iris root. [10] Laser iridotomy carries a risk of IOP spikes, intraocular inflammation, and pigment dispersion. Furthermore, laser iridotomy may lead to progression of peripheral anterior synaechiae, especially more so in eyes with plateau iris. [11] This brings forth the question of whether laser iridotomy should be performed in UBM-confirmed cases of plateau iris associated with iridociliary cysts, especially since it may cause more harm than benefit.

Laser iridocystostomy has been described for the treatment of iridociliary cysts causing angle closure glaucoma. [12] In this case report, the areas with the largest cysts were initially pretreated with argon laser followed by Nd:YAG laser to perforate the cysts. Successful perforation of the iris stroma and anterior walls of the cysts was confirmed by the large gush of pigment outflow into the anterior chamber. This procedure similarly carries a risk of IOP spikes, intraocular inflammation, pigment dispersion, and cyst recurrence. It is also not practical in cases with multiple small cysts.

Argon laser peripheral iridoplasty has recently been described as a safe and effective treatment method for plateau iris. [13] When the pupil is constricted, laser burns are applied to the far periphery of the iris, with resultant contraction of iris stroma peripherally and subsequent widening of the iridotrabecular angle. These thermal burns are usually set with large treatment area, long duration, and low energy, and placed circumferentially as close to the limbus as possible. The treatment effect is generally maintained for years, although repeat laser treatments may be required in some patients. [13] Laser peripheral iridoplasty has also been reported to successfully treat plateau-like iris configuration caused by iridociliary cysts. [14] However, following laser peripheral iridoplasty, the iridotrabecular drainage angle is usually not uniformly open, and some areas will still remain appositionally closed.

Our case is unusual in 3 aspects. Firstly, our patient was a middle-aged man, and so he did not fit in with the typical patient profile for plateau iris and iris cysts. Secondly, although the iridociliary cysts were intermittently spread over 360 degrees, they were not found in all meridians. However, the plateau iris anatomy was present over 360 degrees, even where there were no iridociliary cysts seen on UBM. We were therefore unable to ascertain if the iridociliary cysts were indeed causing the plateau iris syndrome, or if these were 2 coincidental findings. Thirdly, there was no improvement in appositional iridotrabecular angle closure despite having had both laser peripheral iridotomy and iridoplasty.

So, what is the best management option for our patient? Should we do nothing and merely observe him? The probability of developing acute or chronic angle closure glaucoma from plateau iris is unknown. What we do know is that our patient remains at risk of acute or chronic angle closure glaucoma, which may occur years down the line. This risk may increase as the lens enlarges with age. Would it be possible to modify this risk? Lensectomy was not considered in our patient as he only had mild lenticular opacities, and still retained good distance and near vision (6/5 and N5) in both eyes. Furthermore, the mechanical presence of the iridociliary cysts suggests that his iridotrabecular angle is unlikely to be widened following lensectomy. It has also been reported that cataract extraction does not alter the plateau iris configuration, although it increases the anterior chamber depth. [15] Low concentration topical pilocarpine is possibly an option, especially if he is able to tolerate the side effects in the long term. It may stretch and thin the peripheral iris sufficiently to relieve any iridotrabecular angle apposition. However, it remains unclear as to how the pilocarpine may affect the iris cysts in the long term.

Our patient is currently not on any anti-glaucoma medication, as his visual fields and optic discs looked healthy. He is currently under close review for gonioscopy, UBM assessment, and IOP monitoring.

## Conclusion

This case illustrates the difficulties in the management for patients with plateau iris in conjunction with iridociliary cysts. Making the diagnosis can be difficult, and whenever possible, UBM should be performed on patients with appositional iridotrabecular angle closure on gonioscopy, especially if the angle closure is not relieved with either laser peripheral iridotomy or iridoplasty. However, question marks can be raised as to the benefit of laser iridotomy when plateau iris without pupillary block has already been conclusively diagnosed on UBM.

## Abbreviations

Abbreviations used in the text have been defined in the text where first used.

## Consent

Written informed consent was obtained from the patient for publication of this case report and accompanying images. A copy of the written consent is available for review by the Editor-in-Chief of this journal.

## Competing interests

The authors declare that they have no competing interests.

## Authors' contributions

GSA, FB and AAB analyzed and interpreted the patient data regarding the ocular examination. FB performed the ultrasound biomicroscopic assessment. All authors were major contributors in writing the manuscript. All authors read and approved the final manuscript.
